# The Co-Expression Pattern of Odorant Binding Proteins and Olfactory Receptors Identify Distinct Trichoid Sensilla on the Antenna of the Malaria Mosquito *Anopheles gambiae*

**DOI:** 10.1371/journal.pone.0069412

**Published:** 2013-07-05

**Authors:** Anna Schultze, Pablo Pregitzer, Marika F. Walter, Daniel F. Woods, Osvaldo Marinotti, Heinz Breer, Jürgen Krieger

**Affiliations:** 1 University of Hohenheim, Institute of Physiology, Stuttgart, Germany; 2 Developmental Biology Center, University of California Irvine, Irvine, California, United States of America; 3 Inscent Inc., Irvine, California, United States of America; Plant and Food Research, New Zealand

## Abstract

The initial steps of odorant recognition in the insect olfactory system involve odorant binding proteins (OBPs) and odorant receptors (ORs). While large families of OBPs have been identified in the malaria vector *A. gambiae*, little is known about their expression pattern in the numerous sensory hairs of the female antenna. We applied whole mount fluorescence in Situ hybridization (WM-FISH) and fluorescence immunohistochemistry (WM-FIHC) to investigate the sensilla co-expression of eight *A. gambiae* OBPs (AgOBPs), most notably AgOBP1 and AgOBP4, which all have abundant transcripts in female antenna. WM-FISH analysis of female antennae using AgOBP-specific probes revealed marked differences in the number of cells expressing each various AgOBPs. Testing combinations of AgOBP probes in two-color WM-FISH resulted in distinct cellular labeling patterns, indicating a combinatorial expression of AgOBPs and revealing distinct AgOBP requirements for various functional sensilla types. WM-FIHC with antisera to AgOBP1 and AgOBP4 confirmed expression of the respective proteins by support cells and demonstrated a location of OBPs within sensilla trichodea. Based on the finding that AgOBP1 and AgOBP4 as well as the receptor type AgOR2 are involved in the recognition of indole, experiments were performed to explore if the AgOBP-types and AgOR2 are co-expressed in distinct olfactory sensilla. Applying two-color WM-FISH with AgOBP-specific probes and probes specific for AgOR2 revealed a close association of support cells bearing transcripts for AgOBP1 and AgOBP4 and neurons with a transcript for the receptor AgOR2. Moreover, combined WM-FISH/-FIHC approaches using an AgOR2-specific riboprobe and AgOBP-specific antisera revealed the expression of the “ligand-matched” AgOBP1, AgOBP4 and AgOR2 to single trichoid hairs. This result substantiates the notion that a specific response to indole is mediated by an interplay of the proteins.

## Introduction

**Figure 1:**
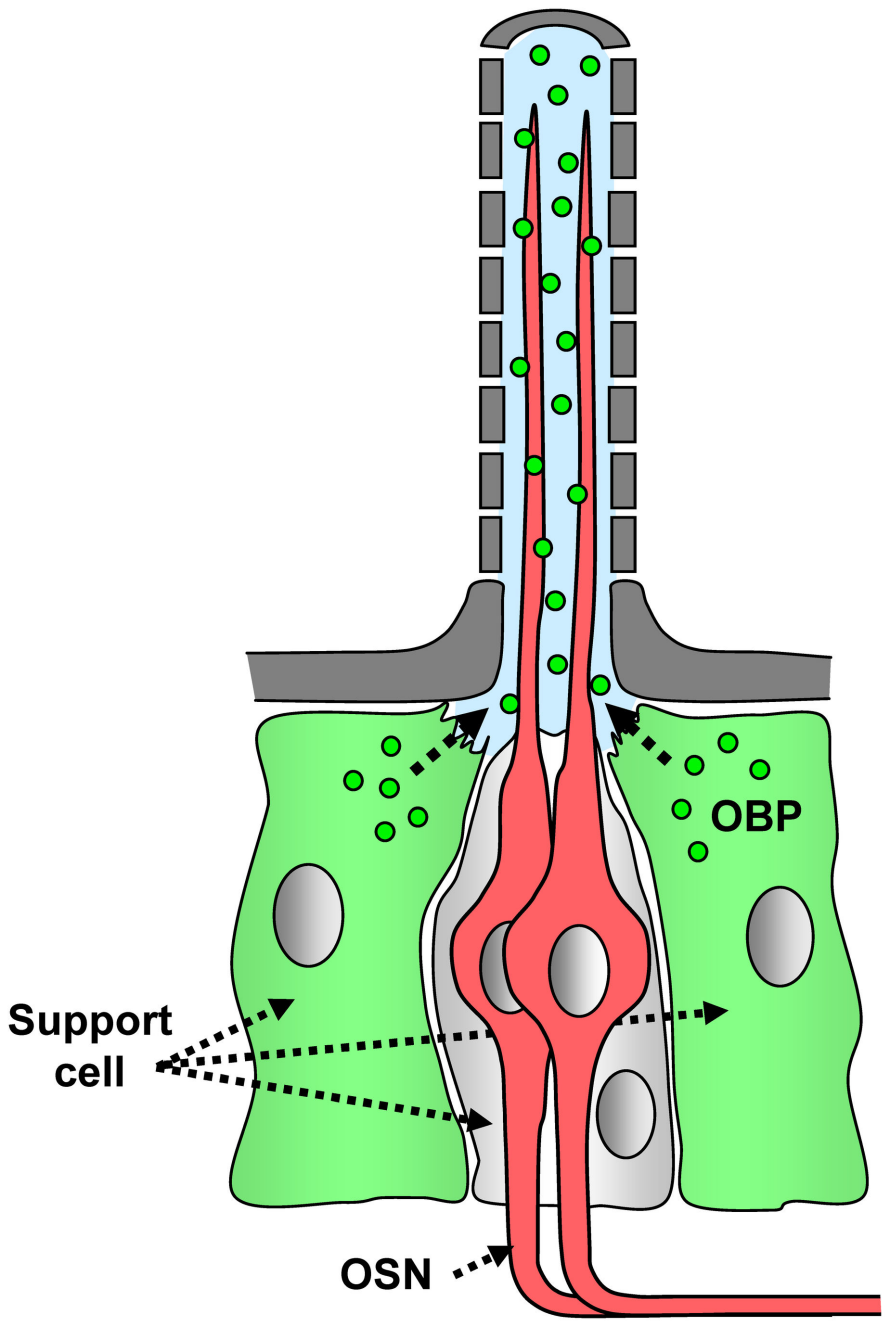
General organization of a trichoid sensillum hair. Two olfactory sensory neurons (OSNs, red) project their dendrites into the sensillum lymph (blue). The cell bodies of the OSNs are surrounded by three support cells, two of which express “classic” odorant binding proteins (OBPs, dark green) and secret them into the sensillum lymph.

The mosquito *Anopheles gambiae* is a major vector for several human pathogens, which affect millions of people in afrotropical regions by causing the life-threatening disease malaria as well as human filariasis and O’nyong-Nyong fever [1–3]. The transfer of pathogenic parasites or virus is mediated solely by blood-feeding female mosquitoes, which depend on a protein-rich blood meal to complete their gonadotrophic cycle, but otherwise feed on nectar like the males. Female mosquitoes are predominantly guided by olfactory cues to blood hosts, nectar sources, and oviposition sites [4,5]. In female *A. gambiae* volatile odors emitted from humans, plants or stagnant water are detected by their principal olfactory organs, the antennae. Each antenna has about 1500-1600 olfactory sensory neurons (OSNs) that are housed in around 730 hair-like compartments, called sensilla (Figure 1), mainly of the trichoid type [6,7]. A few olfactory sensilla are also found on the maxillary palps and the proboscis that contain OSNs that respond to plant-derived volatiles and human-related odorants, including carbon dioxide [8,9].

Numerous studies on various insects [10–13] have indicated that the detection of odorants by the antenna involves specific odorant receptors (ORs) in the membrane of OSNs as well as odorant binding proteins (OBPs) in the aqueous sensillum lymph bathing the receptive dendrites of OSNs (Figure 1). The *A. gambiae* genome contains about 60 genes encoding putative OBPs [14–18] and a repertoire of 76 genes encoding ORs [17,19–21]. In support of their role in olfaction, transcripts for the majority of AgOR genes [17,22] and many putative AgOBPs [17,23] have been identified in the antennae and maxillary palps. Several *A. gambiae* ORs and OBPs show higher levels of expression in female antenna and altered transcript levels after a blood meal. These AgORs and AgOBPs are therefore considered as particularly important for the detection of odorants emitted from blood hosts or oviposition sites [17,20,22–24]. Very high transcript levels have been found for several “classic” AgOBPs (with the highly conserved pattern of six cysteines) as well as for two “Plus-C” AgOBPs (with a longer primary structure and additional cysteines) in female antenna [17,23]. WM-FISH studies have shown that the gender difference in transcript levels of AgOBP1 and the “Plus-C” AgOBP48 reflects a marked difference in the number of cells expressing these genes, with many more cells in females compared to males [25,26]. Similarly, higher transcript levels were found for several odorant receptors [22], and this corresponds to a higher number of expressing cells in female antenna [26].

Specific roles of the various ORs in *A. gambiae* olfaction have been indicated from studying the binding specificities of a total of 50 ORs expressed in *Xenopus* oocytes [27] and the “empty neuron” system of *Drosophila* [28,29]. For different AgORs response profiles and variable tuning breadths have been found. For example, AgOR1 was found to be rather narrowly tuned to 4-methylphenol a mosquito attractant volatile found in human sweat [29]. Similarly, AgOR2 responded to a narrow set of odorants [27,28] with indole as the best ligand, an odorant released from blood hosts or water from breeding sites of *A. gambiae* [30,31]. Ligand binding spectra have also been determined for a number of AgOBP types. Using a 1-NPN-based competitive binding assay diverse and partly overlapping odorant binding specificities were found [32]. With regard to a possible interplay of AgOBPs and AgORs in the detection of odorants, the available binding spectra of both protein types thus far show little similarity. However, an interesting “ligand-match” of two AgOBPs and an AgOR was found for indole. Indole activates quite specifically the OR2 of *A. gambiae* [27,28–28] and its orthologues in other mosquitoes [33,34]. A crucial role for the AgOBP1 and AgOBP4 binding proteins in indole detection was demonstrated by RNAi-based gene silencing, binding studies and structural analysis [35,36].

So far, little is known whether different AgOBP-subtypes are co-expressed in individual sensilla, especially those which are predicted to form OBP heteromers [32,36,37]. In this study we have examined the cellular expression of eight AgOBPs (seven “classic” AgOBPs and AgOBP48); all of them had high levels of expression in female antenna. Furthermore, we explored if the OBP-subtypes AgOBP1 and AgOBP4 and the receptor subtype AgOR2, which are considered as “ligand-matched” proteins for indole, are co-expressed within the same sensillum hair.

## Materials and Methods

### Animal rearing

Larvae of the *Anopheles gambiae* s.s. strains “Kisumu” and “RSP” were kindly provided by Bayer CropScience AG, Monheim, Germany. Both laboratory strains were originally derived from the region of Kisumu, Kenya. Animals developed to adults at 28°C with a day/night cycle of 12:12. After emergence, animals had access to 10% sucrose *ad libitum*. Antennae for *in situ* hybridization experiments were dissected from adult females within 3 days of emergence.

### Preparation of labeled probes for *in situ* hybridization

Digoxigenin (DIG)-labeled and biotion-labeled antisense RNA probes as well as DIG-labeled sense RNA probes for *in situ* hybridization were generated from linearized pGem-T Easy and pBluescript plasmids containing the coding regions of AgOBP and AgOR genes (Tab. S1). For *in vitro* transcription the SP6/T3/T7 RNA transcription system (Roche) was used following recommended protocols. To improve tissue penetration labeled riboprobes were partially fragmented to an average length of about 200 bp by incubation in carbonate buffer (80 mM NaHCO_3_ 120 mM Na _2_CO_3_, pH 10.2) following the protocol of [38].

### Double whole mount fluorescence in situ hybridization (WM-FISH)

Two-color *in situ* hybridization with complete antennae and two differentially labeled (DIG or biotin) antisense RNA probes (double WM-FISH) were performed as described earlier [25,32] with few modifications. Incubations and washes were made in a volume of 0.25 ml using thin walled PCR tubes (Kisker, Germany) applying slow rotation or moderate shaking. Antennae were dissected from cold anesthetized animals transferred to a fixation solution (4% paraformaldehyde in 0.1 M NaCO_3_, pH 9.5, 0.03% Triton X-100) and incubated for 20-24 hours at 6°C. After a 1 min wash at room temperature in PBS (phosphate-buffered saline = 0.85% NaCl, 1.4 mM KH_2_PO_4_, 8 mM Na_2_HPO_4_, pH 7.1), 0.03% Triton X-100, antennae were carefully squeezed about ten times with a fine forceps in the same solution under binocular inspection. This treatment caused small cracks in the cuticle and improved penetration of solutions into tissue. Subsequently, antennae were incubated for 10 min in 0.2 M HCl, 0.03% Triton X-100, washed for 1 min in PBS, 1% Triton X-100 and prehybridized at 55°C for at least 6 hours with *in situ* hybridization solution (50% formamide, 5x SSC, 1x Denhardt’s reagent, 50 µg/ml yeast RNA, 1% Tween 20, 0.1% Chaps, 5 mM EDTA pH 8.0). When not used immediately, antennae were stored at 6°C in hybridization solution for a maximum of two days. After prehybridization, antennae were incubated in hybridization solution containing labeled antisense RNA probes at 55°C for at least 48 hours. In control experiments (Figure S1) labeled sense RNA probes instead of antisense RNA probes were used. After washing the antennae four times for 15 min each in 0.1x SSC, 0.03% Triton X-100 at 60°C, 1% blocking reagent (Roche) in TBS (100 mM Tris, pH 7.5, 150 mM NaCl), 0.03% Triton X-100 was added for 5 hours at 6°C. Then DIG-labeled RNA probes were detected by incubating the tissue with an anti-DIG AP-conjugated antibody (Roche) diluted 1:500 in TBS, 0.03% Triton X-100, 1% blocking reagent; for simultaneous detection of biotin-labeled probes a streptavidin horse radish peroxidase-conjugate (1:100, TSA kit, PerkinElmer) was included. After at least 48 hours at 6°C, antennae were washed five times in TBS with 0.05% Tween 20 for 10 min each at room temperature. This was followed by incubation with HNPP (Roche; 1:100 in DAP-buffer (100 mM Tris, pH 8.0, 100 mM NaCl, 50 mM MgCl_2_) for 5-6 hours at 6°C in the dark to visualize DIG-labeled probes. The biotin-labeled probes were visualized after three 5 min washes in TBS with 0.05% Tween 20 by using the TSA kit / FITC development and incubation for 17-18 hours at 6°C in the dark. This step was omitted in WM-FISH with only a DIG-labeled probe. Finally, antennae were washed in TBS with 0.05% Tween 20 three times for 5 min and briefly rinsed in PBS, before they were mounted in mowiol solution (10% polyvinylalcohol 4-88, 20% glycerol in PBS).

### Anti-AgOBP antibodies

For imunolocalization of OBPs in antennae we used a previously described polyclonal antiserum against AgOBP1 (Biessmann et al., 2010) and generated a polyclonal antibody against AgOBP4 in the same way. Briefly, the OBP coding region was PCR amplified and cloned into the pRSET vector (Invitrogen). The recombinant 6x His tagged AgOBPs were expressed from pRSET vectors in *E. coli* BL21 Star (DE3) pLysS cells (Invitrogen). After purification of AgOBPs from the soluble fraction of bacterial lysate using the SwellGel Cobalt Chelated Disc system (Pierce Chemical), purified AgOBPs were injected into guinea pigs (Pocono Rabbit Farm Laboratory, Inc., Canadensis, PA), to generate the antibodies.

### Whole mount fluorescence immunohistochemistry (WM-FIHC)

To visualize AgOBPs with the specific antibodies in the antennae we adapted a whole mount fluorescence immunohistochemistry (WM-FIHC) protocol, which includes zinc-formaldehyde (ZnFA) fixation. The protocol is supposed to prevent masking of antigen epitopes and to improve antibody penetration into tissues compared to fixation with paraformaldehyde [39]. If not otherwise stated all incubations and washes were made at room temperature in a volume of 0.25 ml in thin walled PCR tubes (Kisker, Germany) with slow rotation on an overhead shaker.

Antennae were dissected from cold anesthetized animals and transferred directly to ZnFA solution (0.25% ZnCl_2_, 1% formaldehyde, 135 mM NaCl, 1.2% sucrose, 0.03% Triton X-100). After fixation for 16-24 hours at room temperature antennae were washed twice for 15 min each with HBS buffer (150 mM NaCl, 5 mM KCl, 25 mM sucrose, 10 mM Hepes, 5 mM CaCl_2_, 0.03% Triton X-100). Then the antennae were transferred under binocular control to a drop of HBS buffer on a glass slide and carefully squeezed about 10 times with a fine forceps. This was followed by another wash in HBS buffer for 15 min, incubation for 1 hour in 80% Methanol / 20% DMSO and a wash for 5 min in 0.1 M Tris pH 7.4, 0.03% Triton X-100. Subsequently antennae were incubated in blocking solution (PBS, 5% newborn goat serum, 1% DMSO, 0.03% Triton X-100) for at least 3 hours. The solution was replaced by blocking solution containing the primary antibody (dilution 1:100) and the PCR tube was placed for 30 sec in a water bath sonifier (Bansonic 1200, Branson, Danbury, CT). In control experiments pre-immune serum (1:100) instead of the primary antibody was used. Antennae were incubated for 4 days at 6°C, with repeating the sonification on the second day. Washing three times for 15 min each at room temperature in PBS, 1% DMSO, 0.03% Triton X-100 was followed by adding an anti-guinea pig Alexa488 coupled secondary antibody (1:1000) in blocking solution. Sonification for 30 sec in a water bath sonifier was followed by incubation for 3 days at 6°C. After washing three times for 15 min each in PBS with 1% DMSO, 0.03% Triton X-100 and a short rinse in PBS antennae were mounted in mowiol solution.

### Combined WM-FISH and WM-FIHC

If not otherwise indicated handling was at room temperature. Washes and incubations were done under slow rotation on an overhead shaker. Antennae were prepared and fixed in the same way as described for WM-FIHC. After 24 hours in ZnFA solution, antennae were washed in HBS buffer three times for 15 min each. Within the last washing period antennae were mechanically treated in HBS buffer by using a forceps as described for WM-FIHC. Subsequently antennae were shortly rinsed in PBS, 0.03% Triton X-100 and transferred into *in situ* hybridization solution (see above). If not prehybridized directly, antennae were stored at 6°C until use. Prehybridization of antennae was performed at 55°C for 5–6 hours and followed by incubation for 3 days at 55°C in the same solution containing a DIG-labeled antisense RNA. After washing in 0.1 x SSC, 0.03% Triton X-100 four times for 15 min each, antennae were treated with blocking solution (TBS, 5% normal goat serum, 0.03% Triton X-100) for at least 5 hours. Then blocking solution containing the primary antibody (1:100 -1: 500) and the anti-DIG alkaline phosphatase-conjugated antibody (1:500) (Roche) was added. Subsequently the tubes were placed for 30 sec in a water bath sonifier (Bransonic 1200) and then incubated for 3 days at 6°C. After three washed in TBS, 0.05% Tween 20 for 15 min each, antennae were treated with an anti-guinea pig Alexa488 coupled secondary antibody in blocking solution (1:1000). Sonification for 30 sec in a water bath sonifier was followed by incubation for 3 days at 6°C in the dark (all subsequent steps were made under light protection). Antennae were then washed with TBS, 0.05% Tween 20 three times for 15 min, shortly equilibrated in DAP-buffer, pH 8.0, before DIG-labeled probes were visualized by the incubation with HNPP (1:100 in DAP-buffer) at 6°C for 5-6 hours. Finally, antennae were washed three times in TBS, 0.05% Tween 20 for 5 min, briefly rinsed in PBS and mounted in mowiol solution.

### Analysis of antennae by confocal microscopy

Antenna from WM-FISH experiments were analyzed for epifluorescence using a Zeiss LSM510 Meta laser scanning microscope (Zeiss, Oberkochen, Germany). Confocal image stacks of the red and green fluorescence channel as well as the transmitted-light channel were recorded from antennal segments. Image stacks were used to generate pictures representing projections of selected optical planes, with the red and green fluorescence channels overlaid with the transmitted-light channel or shown separately.

## Results

### Expression of OBPs in the antenna of female *A. gambiae*

**Figure 2:**
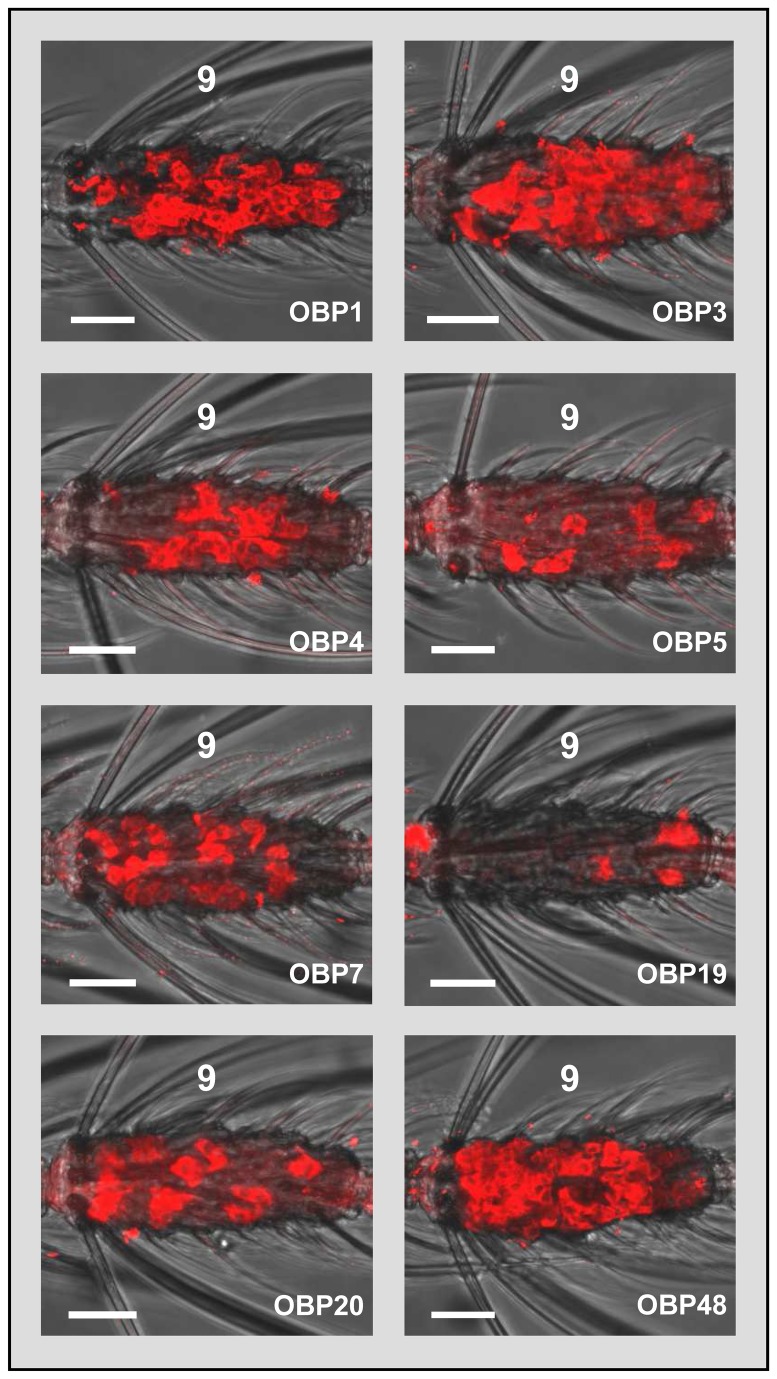
Gene expression of “classic” OBPs and the “Plus-C” OBP48 in the antenna of *A. gambiae* females. WM-FISH using AgOBP-specific DIG-labeled antisense RNA probes. Cells bearing AgOBP transcripts have been visualized by red fluorescence. The same (9th) flagellomere from different animals is shown. Each AgOBP is expressed in either a high, moderate or low number of cells distributed in the antennal segment. Scale bars: 20 µm.

To investigate the combinatorial expression of AgOBPs in the female antenna we selected eight AgOBPs (seven “classic” and one “Plus-C” AgOBP) based on their abundant and significantly enhanced transcript levels in females compared to males as previously indicated by RT-PCR experiments, microarray studies and whole transcriptome RNA sequencing [17,23]. In WM-FISH experiments we first examined the number and topography of the cells expressing the AgOBP genes. Using labeled antisense RNA probes we detected hybridization signals characteristic for labeling of support cells as reported earlier [25,26]. Similar control experiments with labeled sense RNA probes revealed no labeling confirming the specificity of the WM-FISH signals (Figure S1). Although it was not possible to determine the exact number of AgOBP-expressing cells, comparing the hybridization signals in the same antennal segments revealed clear differences between various AgOBPs. We found a striking higher number of AgOBP3-positive cells (Figure 2) and in agreement with previous results [25,26] also for AgOBP1 and AgOBP48. In comparison much lower numbers of cells expressed AgOBPs 4, 5, 7, 19 and 20. For each of the eight AgOBPs tested, expressing cells were detected in antennal segments 3 to 13 (data not shown), with conspicuously higher numbers of labeled cells per segment in more distal segments, likely reflecting the increase in the number of olfactory hairs towards the antennal tip [6]. Cells with transcripts for AgOBP7, AgOBP20 and AgOBP48 were also detected in segment 2. None of the AgOBPs tested were expressed in cells in antennal segment 1. No differences were found between the *A. gambiae* strains “Kisumu” and “RSP”.

### Co-expression pattern of AgOBPs in female antenna

**Figure 3:**
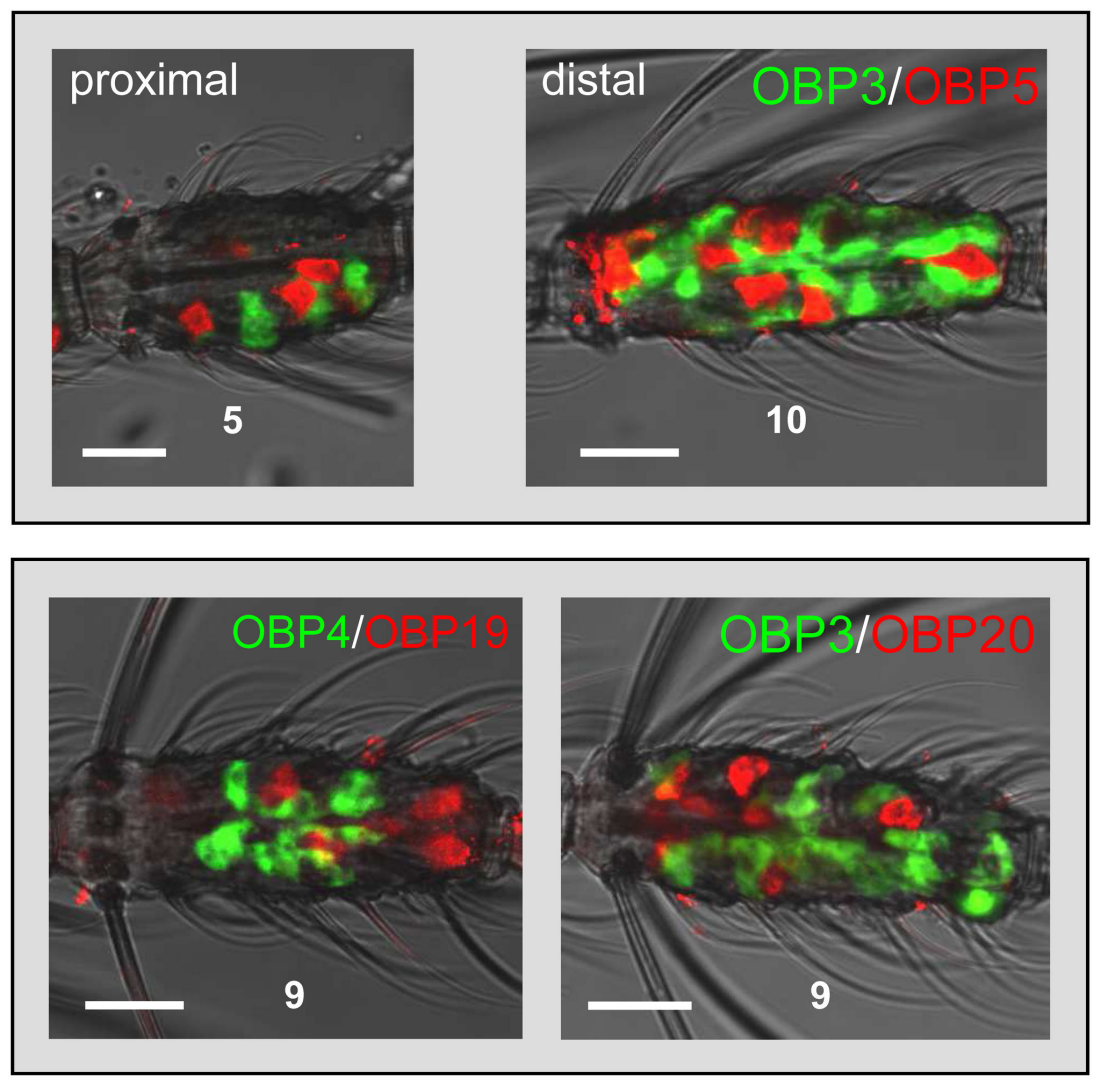
Expression of AgOBP pairs in different cells of the female antenna. Two-color WM-FISH using combinations of differentially labeled antisense RNAs and detection systems visualizing expression of the two AgOBPs by green and red fluorescence, respectively. No co-labeling of single cells is visible indicating expression of the pairs in different cell populations. This labeling pattern was independent of a more proximal or distal position (upper row) of the antennal segment. The number of each antennal segment is indicated. Scale bars: 20 µm.

**Figure 4:**
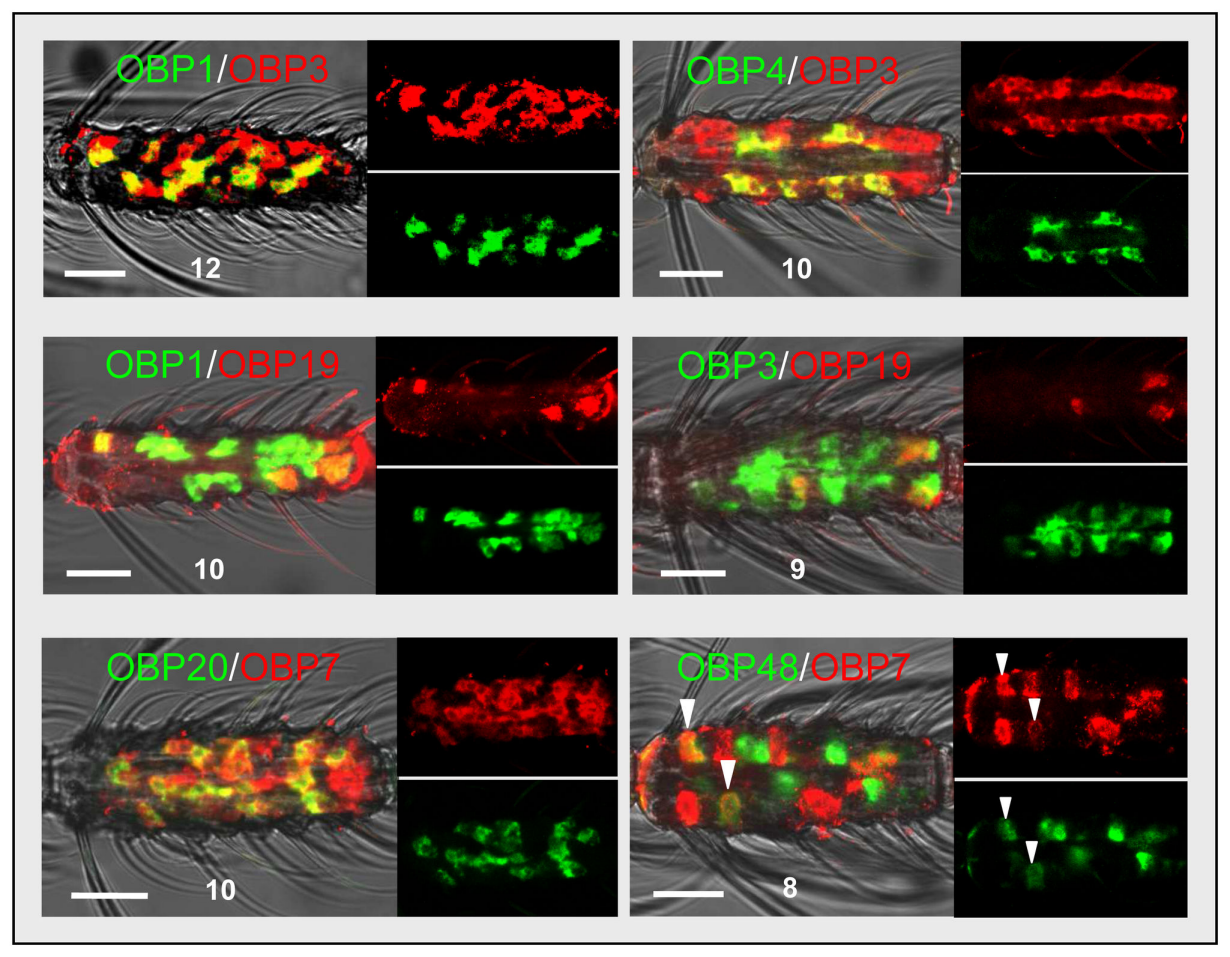
Co-expression of AgOBP pairs in subpopulation of antennal cells. Two-color WM-FISH with female antennae and visualization of cells bearing distinct AgOBP transcripts by green and red fluorescence, respectively. Labeling of the same cells by both AgOBP probes is shown for six pairings. In the case of strong hybridization signals co-labeling appears as yellow color in the overlay (left) of the red and green fluorescence channels (right). For the pair AgOBP7/AgOBP48, which displayed rather weak signals co-labeled cells are marked by arrow heads. The separated fluorescence channels (right) are shown in reduced size. Numbers indicate the position of the antennal segment. Scale bars: 20 µm.

In order to evaluate the co-expression of AgOBPs in female antenna, we applied two-color WM-FISH. Antennae were probed with pairs of differentially labeled AgOBP-specific riboprobes, cells containing transcripts for each AgOBP were visualized by red or green fluorescence, respectively (Figures 3 and 4). Similar to results obtained with single probes, double WM-FISH experiments revealed differences between AgOBPs regarding the number of labeled cells within an antennal segment (Figure 3). As shown for the pairs AgOBP3-AgOBP5, AgOBP3-AgOBP20 and AgOBP4-AgOBP19, most AgOBP combinations tested (22 out of 29, Tab. 1) led to clearly distinguishable red or green labeled cells, indicating expression of each AgOBP in different cell populations. This labeling pattern was independent from the distal or proximal position of the segment (Figure 3). The same results were also obtained for the pairs AgOBP1-AgOBP48 and AgOBP4-AgOBP48 (not shown), thus extending and confirming previous data [25].

**Table 1:** Co-expression of AgOBP pairs.

**OBP1**							
**+++**	**OBP3**						
**+++**	**+++**	**OBP4**					
**―**	**―**	**―**	**OBP5**				
**―**	―	**―**	**―**	**OBP7**			
**+++**	**+++**	**―**	**―**	**―**	**OBP19**		
**―**	**―**	**―**	**―**	**+++**	**―**	**OBP20**	
**―**	**―**	**―**	**―**	**+**	**―**	**―**	**OBP48**

+++ Co-expression in a high number of cells

+ Co-expression in few cells

– No Co-expression found

For seven AgOBP pairings (Tab. 1 Figure 4) we found cells labeled by both probes indicating co-expression of the two AgOBPs. In cases of strong hybridization signals, as obtained for the pairs AgOBP1-AgOBP3, AgOBP3-AgOBP4, AgOBP1-AgOBP19, AgOBP3-AgOBP19 and AgOBP7-AgOBP20 (Figure 4) co-labeling is immediately obvious by the appearance of the yellow color in an overlay of the red and green fluorescence channels. For weaker hybridization signals, co-labeling of cells was verified by careful inspection of the hybridization signals in the separated fluorescence channels (AgOBP7/AgOBP48; Figure 4, arrowheads).

The total number and relative fraction of cells with transcripts for both OBPs varied for different AgOBP pairings. For the combination AgOBP1-AgOBP3 (Figure 4) a very high number of double-stained cells were found, with all AgOBP1 cells (green) also labeled by the AgOBP3 probe (red). A lower number of co-labeled cells were observed for the pairs AgOBP1-AgOBP4 [25]) and AgOBP3-AgOBP4 (Figure 4), with all AgOBP4 cells being also positive for both AgOBP1 and AgOBP3. Therefore a subset of antennal support cells expresses all three AgOBPs. Similarly, the results for the pairs AgOBP1-AgOBP19 and AgOBP3-AgOBP19 demonstrate that all AgOBP19-positive cells also have transcripts for AgOBP1 and AgOBP3. In experiments with AgOBP7-AgOBP20 we found that all AgOBP20 cells also have AgOBP7 transcripts, but that additional cells express AgOBP7. For the pair AgOBP7-AgOBP48 we noted co-expression of both AgOBPs in only a very small fraction of the cells transcribing each AgOBP.

Together, our two-color WM-FISH experiments visualized a mosaic-like, partly overlapping expression pattern of the eight AgOBPs in support cells of the female antenna, suggesting a high number of diverse sensilla types which are equipped with distinct subsets of AgOBPs.

### Antennal co-expression of AgOBP1, AgOBP4 and AgOR2 in distinct sensilla hairs

**Figure 5:**
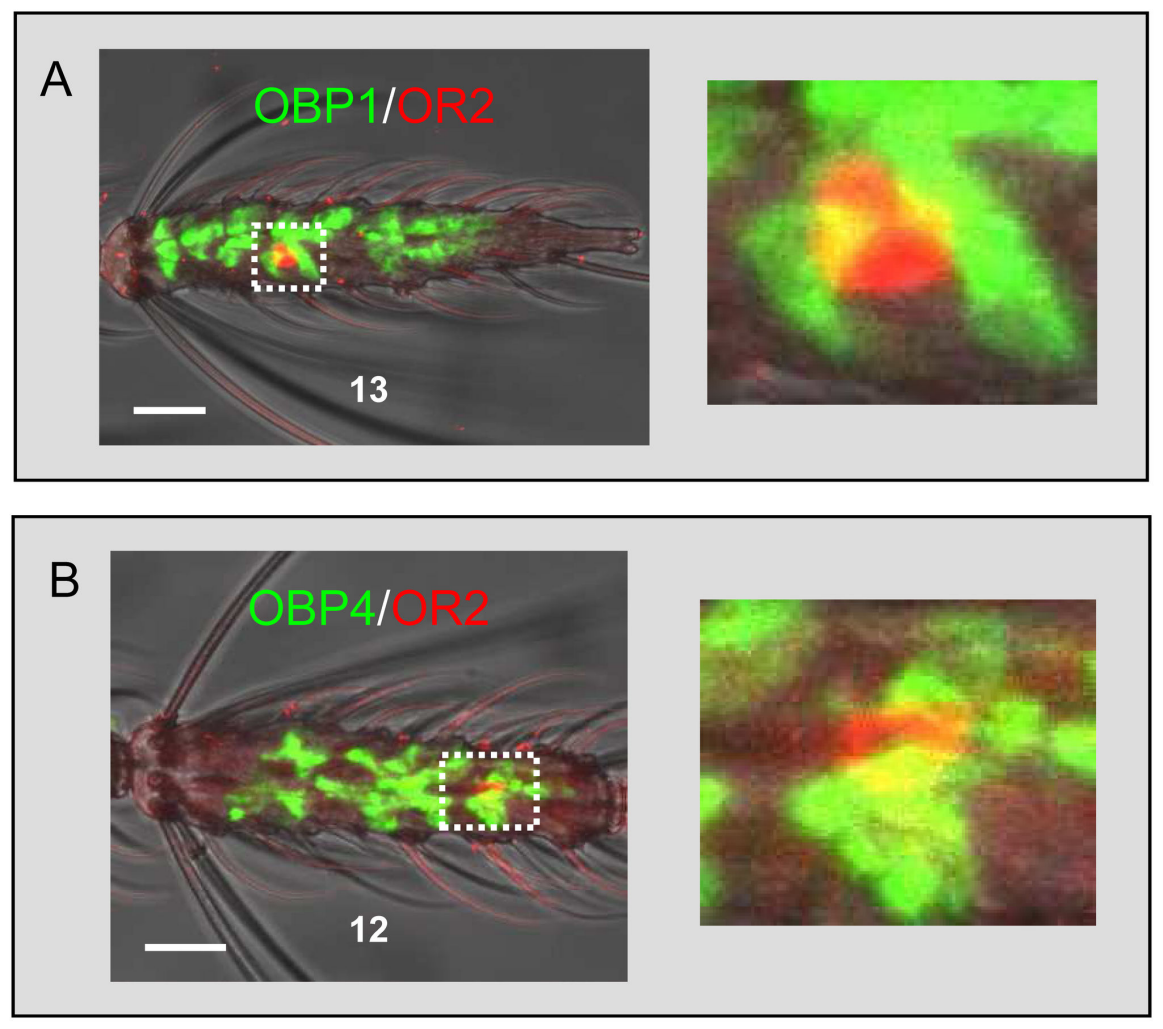
Direct vicinity of AgOR2- and AgOBP-expressing cells. Spatial organization and relative localization of cells transcribing AgOR2 and AgOBP1 (A) and AgOBP4 (B) on female antenna. Double WM-FISH using differentially labeled antisense RNA probes, with visualization of AgOR2 by red and AgOBPs by green fluorescence (Boxed areas are shown at higher magnification on the right). Scale bars: 20 µm.

Finding transcription of the AgOBP1 and AgOBP4 genes in the same cells is in line with a proposed role of homo- and heteromers in indole detection [35–37]. Likewise the olfactory receptor AgOR2 has been reported to mediate specific responses to indole [27,28–28] implying that the receptor and the two “ligand-matched” AgOBPs may cooperate in indole detection. To support or reject this notion we examined whether the cells that express AgOBP1 and AgOBP4 and the cells that express AgOR2 are co-localized in the same sensillum (Figure 5) using two-color WM-FISH. In these experiments we regularly detected an AgOR2-transcribing OSN (red–labeled) in close association with support cells (labeled in green) having transcripts for AgOBP1 (Figure 5A) or AgOBP4 (Figure 5B), indicative of co-localization of the AgOR- and AgOBP-expressing cells in the same sensillum.

**Figure 6:**
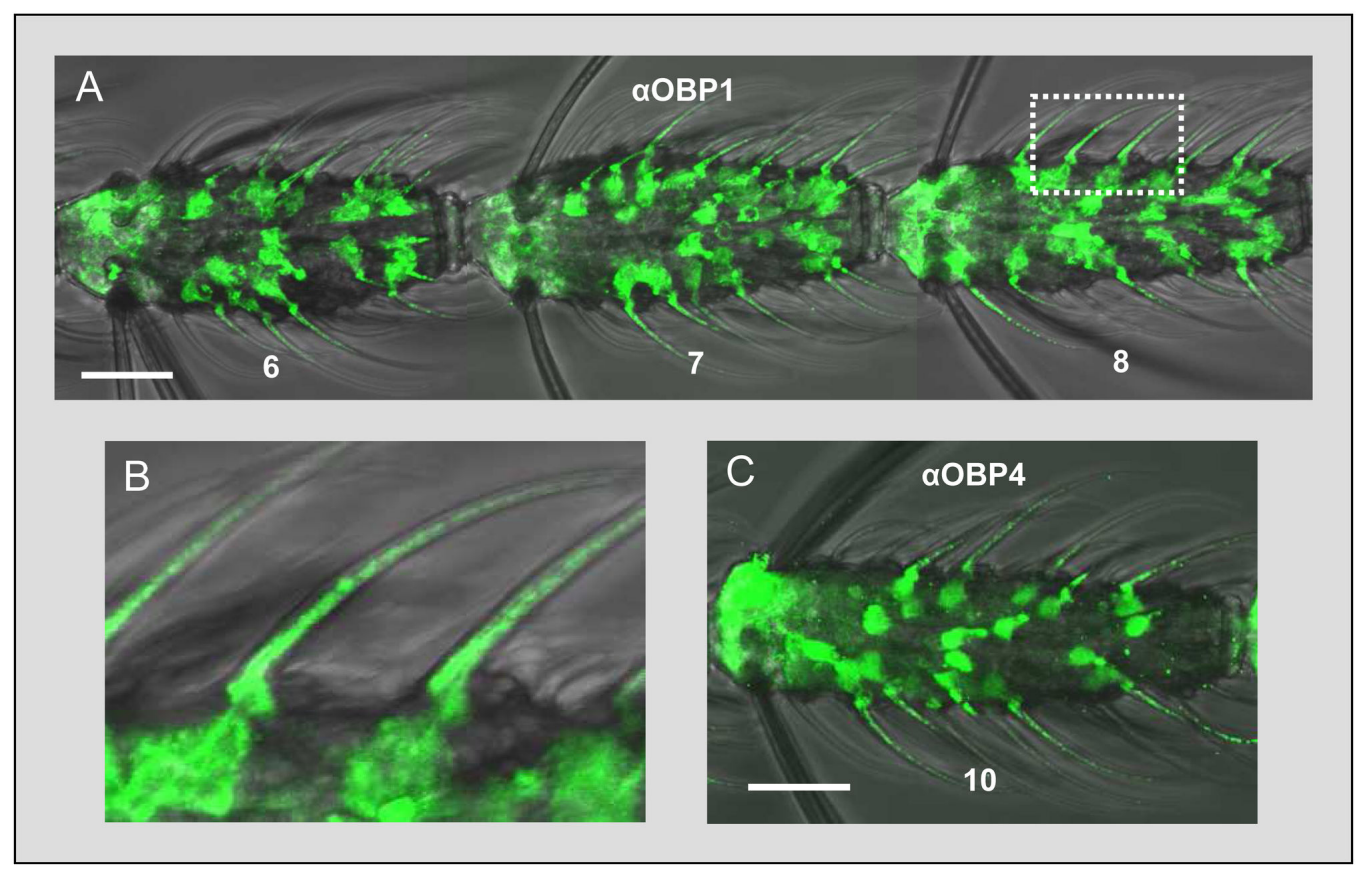
Immunolocalization of OBPs in the antenna of female *A. gambiae*. Whole mount preparations were probed with antisera specific for AgOBP1 (A and B) or AgOBP4 (C). Immunoreactivity was visualized by an Alexa488 secondary antibody. (A) Staining by AgOBP1 antibodies in consecutive antennal segments (6 to 8). A high number of AgOBP-expressing cells is visible and can be assigned to sensilla trichodea. (B) Higher magnification of the area boxed in A. AgOBP1 immunostaining is found within the sensilla hairs as well as in supporting cells below the sensilla. (C) Staining of AgOBP4-expressing cells and sensi lla in antennal segment 10. The antennal stretch shown in (A) was assembled from three single pictures. Scale bars: 20 µm.

To allow a clearer assignment of sensilla co-localization and identification of the sensillum type housing AgOBP1, AgOBP4 and AgOR2 we applied antisera generated against AgOBP1 and AgOBP4 in WM-FIHC experiments. OBP immunoreactivity was visualized as green fluorescence using a secondary Alexa488-coupled antibody and confocal laser scanning microscopy (Figure 6). With the AgOBP1 antiserum (Figure 6A) we obtained intensive labeling under long sensilla hairs. Similar control experiments with the respective pre-immune serum produced no labeling (Figure S2). At higher magnification (Figure 6B) green staining was found in cells below the base of the sensilla hair shafts and in addition could be detected within the sensilla hairs and based on their size and morphology were classified as sensilla trichodea [6]. Not all sensilla trichodea were labeled by the antiserum; in addition no labeling of other sensilla types (grooved pegs, sensilla coeliconica and sensilla chaetica) was noted. A similar staining pattern was obtained in WM-FIHC with antibodies specific for AgOBP4 (Figure 6C). In concurrence with the results from WM-FISH experiments employing AgOBP-specific riboprobes (Figures 2-4). The number of labeled sensilla trichodea appeared lower for anti-AgOBP4 compared to anti-AgOBP1.

**Figure 7:**
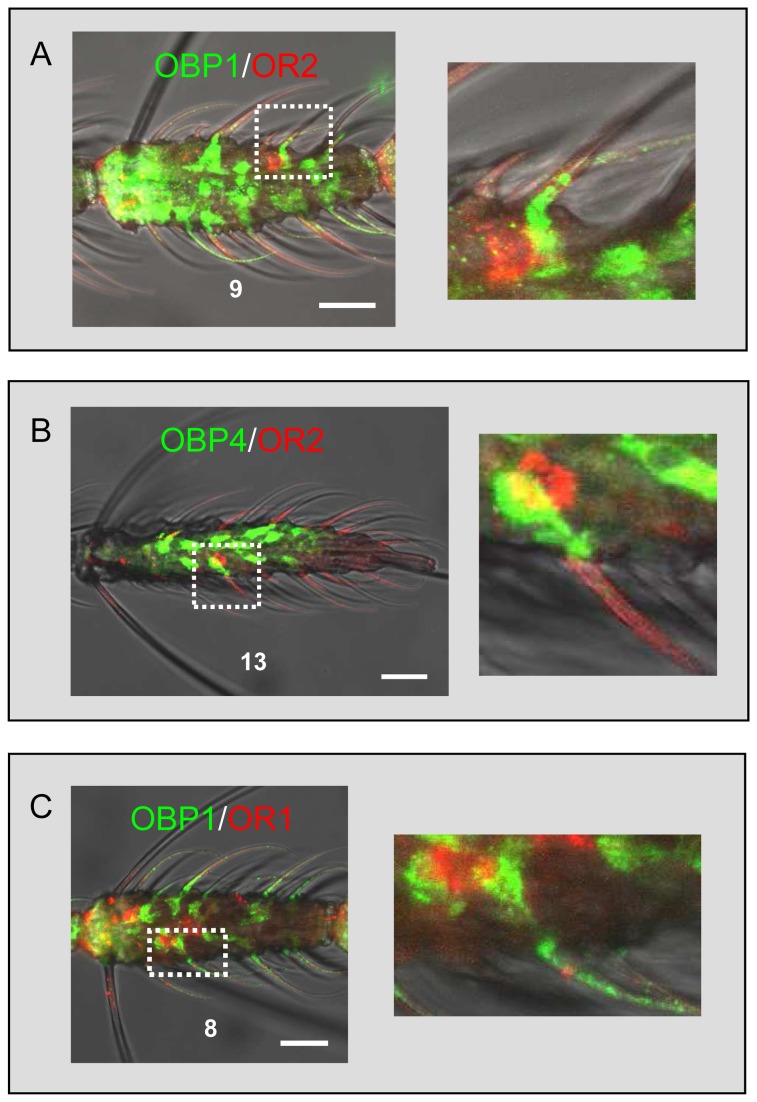
Co-localization of AgOBP and AgOR expression in single sensilla of female antenna. Combination of WM-FIHC using AgOBP-specific antibodies and WM-FISH employing AgOR-specific DIG-labeled antisense probes. (A) AgOBP1 and AgOR2, (B) AgOBP4 and AgOR2, (C) AgOBP1 and AgOR1. OBP-immunostaining (green) is found in direct association with a red labeled OR-transcribing cell (left pictures) and can be followed into a distinct sensillum (boxed areas shown at higher magnification on the right). Scale bars: 20 µm (left).

Due to the lack of AgOR2-specific antibodies, we could not apply two-color WM-FIHC to simultaneously visualize the AgOR2 and AgOBP proteins and verify co-localization of the proteins in single sensilla. As an alternative, we established a combined WM-FISH/WM-FIHC method using an AgOR2-specific riboprobe and the AgOBP-specific antisera. In WM-FISH/WM-FIHC experiments with the combination AgOR2-AgOBP1 (Figure 7A) the anti-AgOBP1 staining pattern was similar to the immunolabeling obtained in single WM-FIHC (Figure 6A), with labeling of many support cells per segment and visualization of AgOBP1 within the lumen of trichoid hairs. In addition AgOR2-expressing cells were visualized by the riboprobe (Figure 7A), resembling the scattered AgOR2 expression pattern previously reported [26]. By inspecting the overlay of the FISH- and FIHC-signals at higher magnification it is obvious that the cell body of the AgOR2-transcribing OSN is in immediate vicinity to the green AgOBP1 antiserum staining. The AgOBP1 staining can be followed into a distinct trichoid hair, thus allowing clear assignment of the AgOR2-expressing cells to the same sensillum as AgOBP1. Similarly, experiments using the AgOR2 riboprobe and the anti-AgOBP4 antiserum demonstrated a co-localization of the AgOR2-expressing OSN and the AgOBP4 protein in sensilla trichodea (Figure 7B).

Our data demonstrate that a subpopulation of the antennal sensilla trichodea is equipped with AgOBP1 and AgOBP4. Of these only a very small fraction of trichodea contains OSN expressing AgOR2, indicating that in the remaining trichoid hairs the three AgOBPs are co-expressed with other AgORs. In agreement with this conclusion previous WM-FISH experiments have found that AgOBP1- and AgOBP4-transcribing cells are closely associated with OSNs containing AgOR1-transcripts [25]. To extend these results and to confirm the co-existence of AgOR1 and the AgOBP proteins within the same trichoid sensillum we performed WM-FISH/WM-FIHC combining an AgOR1-specific riboprobe and anti-AgOBP1 or anti-AgOBP4 antibodies, respectively. These results strongly indicate that AgOBP1 (Figure 7C) as well as AgOBP4 (data not shown) co-exist in the sensillum lymph of a trichoid hair, that houses the dendrite of an AgOR1-expressing OSN.

## Discussion

The results of this study indicate that out of the investigated “classic” AgOBPs and one “Plus–C” class AgOBP some proteins are pair wise co-expressed in the same sensillar hair of the female antenna from *A. gambiae*. We found that the same AgOBP types were combined with different AgOBP subtypes in distinct sensilla. Overall, the WM-FISH experiments uncovered a complex pattern of AgOBP expression, indicating that various sensilla types on the female mosquito antenna have a distinct or partially overlapping OBP milieu. This topography of AgOBP expression further supports the view that OBPs are not just simple general solubilizers and transporters for odorants but in addition make a decisive contribution to odorant recognition [11,13,40]. Considering the overlapping and rather broad ligand binding-spectra of OBPs from *A. gambiae* [32] as well as the response spectra of electrophysiological characterized sensilla types on the mosquito antenna [7], it is conceivable that a single OBP may contribute to the detection of several odorants. Conversely, different OBPs may also be involved in the recognition of a specific compound. Such a scenario is corroborated by the results of a comprehensive analysis of 17 *Drosophila* OBPs. Applying RNAi-mediated suppression of OBP expression and monitoring the behavioral response of flies to ecologically relevant odorants it was found that silencing a distinct OBP altered the response of flies to a subset, but not all odorants. In addition, the response to a specific odorant was affected by attenuating the expression of several OBPs [13].

Here we investigated a subset of seven “classic” AgOBPs and one of the three “Plus-C” AgOBPs that are transcribed in the female antenna [17,23]. The observation that OBPs, such as AgOBP5, which appeared not to be co-expressed with any other AgOBP type does not exclude the possibility that AgOBP5 is co-localized with a different AgOBP type, that has not been tested in this study. Also, in cases where the transcripts of two OBPs are not found in the same cell by *in situ* hybridization does not excluded the possibility that both proteins are present within a sensillum; the two OBPs could be expressed and secreted by different support cells housed in the same sensillum. This possibility was recently substantiated by a study determining the relative location of cells expressing AgOBP1 and AgOBP48 and the receptor AgOR1. It was demonstrated that AgOBP1 was expressed by two of the three support cells in the sensillum, which houses the AgOR1-expressing OSN, whereas AgOBP48 was expressed by the third cell [25].

Co-expression of general OBPs, pheromone binding proteins (PBPs) or combinations of both types in the same sensillum has been shown for a number of moths species [41–44] and the fruit fly *Drosophila melanogaster* [13,45,46]. It is particularly interesting to note that some of the OBPs analyzed here have a similar topographic expression pattern as their counterparts in *Drosophila melanogaster*. OBP1 and OBP3 from *A. gambiae* are orthologues of the *Drosophila* OBP types OS–F and OS-E, respectively [16,47,48], and AgOBP4 shares high sequence similarity with the PBP LUSH [32,48]. We have found that AgOBP1 and AgOBP3 are broadly expressed in sensilla trichodea of *A. gambiae* and co-expressed with AgOBP4 in a large fraction of trichoid hairs. Similarly, in *Drosophila* LUSH and the OBPs OS-E and OS–F are housed together in trichoid sensilla and in addition OS-E and OS–F are expressed in sensilla intermedia [45,46,49]. Overall, these findings demonstrate a similar combination of phylogenetically related OBPs in antennal sensilla of the mosquito *A. gambiae* and the fruitfly *Drosophila* and may suggest that in the dipteran lineage the OBP equipment of certain trichoid sensilla may have been conserved during evolution.

The specific functional roles of the orthologous OBPs from *Anopheles* and *Drosophila* in olfaction is still unclear but for the related binding protein LUSH it has convincingly been shown that it is essential for detection of the pheromone 11-cis-vaccenyl acetate in trichoid sensilla [50,51], while ligands for the binding proteins OS-E and OS–F are unknown. Competitive binding studies have shown that AgOBP1, AgOBP4 and AgOBP3 share some similarities in their ligand spectra and it has been argued that AgOBP1 and AgOBP3 may cooperatively interact with AgOBP4 [32]. In addition, AgOBP1–AgOBP4 heteromers have been proposed based on co-immunoprecipitation and protein crosslinking studies [37] suggesting a possible functional relationship between these *Anopheles* proteins.

Two studies reported that both AgOBP1 [35] and AgOBP4 [36] are able to bind indole. Together with the finding that RNAi-based silencing of AgOBP1 expression suppressed the electrophysiological response of the antenna to indole [35], the data indicates a crucial role of AgOBP1 and AgOBP4 in indole recognition. Interestingly, indole is the best ligand for OR2, one of the most narrowly tuned *A. gambiae* odorant receptors (Carey et al., 2010; Wang et al., 2010). This implies that the “ligand-matched” AgOBP1 and/or AgOBP4 may mediate the transfer of indole to AgOR2. We have found single AgOR2-expressing OSNs tightly associated with AgOBP1- and AgOBP4-expressing support cells and could clearly assign both AgOBPs and the AgOR2-expressing cell to the same trichoid sensillum. Therefore, our results are in agreement with the concept that AgOBP1 and AgOBP4 may functionally cooperate with AgOR2 and mediate a sensitive and specific recognition of indole. Our data demonstrate for the first time co-localization of “ligand-matched” OR and OBPs in *A. gambiae* olfactory sensilla, a scenario which is reminiscent of the organization of pheromone-responsive sensilla in lepidopteron species and *Drosophila* [41,42,50–53]. However, unlike the pheromone detection system of moths, where thousands of identically equipped sensilla hairs are used to detect a specific pheromone [54,55] the mosquito olfactory system appears to employ combinations of differentially equipped chemosensory units to recognize odorants of high behavioral relevance. In agreement with such a combinatorial principle of odorant detection, the receptor AgOR2 is expressed only in a very small fraction of the sensilla, which contain both AgOBP1 and AgOBP4. Hence, in other sensilla AgOBP1 and AgOBP4 apparently co-exist with other AgOR types, some of which may also recognize indole. In fact, other *A. gambiae* odorant receptors, for example OR10, respond to indole in heterologous expression systems [27,28].

In conclusion, our results indicate a complex topographic pattern for OBP- and OR-expression in antennal sensilla of female *A. gambiae*. The combinatorial arrangement of different AgOBP- and AgOR-subtypes in sensilla may form the molecular basis for the ability of mosquitoes to detect and discriminate relevant olfactory cues within the vast spectrum of odorants originating from vertebrate hosts, oviposition sites or food plants [56,57].

## Supporting Information

Figure S1 WM-FISH with OBP- and OR-specific sense RNA probes revealed no hybridisation signals. WM-FISH using AgOBP- or OR-specific DIG-labeled sense RNA probes and female *A. gambiae* antennae. No fluorescence labeling of cells (which would appear as red staining) was obtained with any of the sense probes tested. Only very weak background staining was obtained in some cases. The same (9th) flagellomere from different animals is shown. Pictures were taken using the same laser scanning microscope settings as the pictures shown in Figure1 for the OBP antisense RNA probes Scale bars: 20 µm.
(TIF)

Figure S2 WM-FIHC with pre-immune serum from OBP1-immunized animals revealed no immunolabeling of cells. Whole mount preparations of female antennae were probed with pre-immune antiserum from animals, which were used to generate the OBP1-specific antiserum. No labeling of cells (which would be indicated by green color) was obtained with the pre-immune serum. The numbers of the flagellomere shown is indicated. Pictures were taken using the same laser scanning microscope settings as the pictures shown in Figure 6 for the OBP1 antiserum. Scale bars: 20 µm.
(TIF)

Table S1 AgOBP- and AgOR-sequences used for WM-FISH. AgOBPs and AgORs Gene Bank Accession numbers and nucleotide regions used as probes in whole mount fluorescence *in situ* hybridization experiments are indicated.
(DOC)
