# Supplementary figures and images for: The Co-Expression Pattern of Odorant Binding Proteins and Olfactory Receptors Identify Distinct Trichoid Sensilla on the Antenna of the Malaria Mosquito Anopheles gambiae
Source: PLoS One. 2013 Jul 5;8(7):e69412. doi: 10.1371/journal.pone.0069412 (PMC3702612; doi:10.1371/journal.pone.0069412)

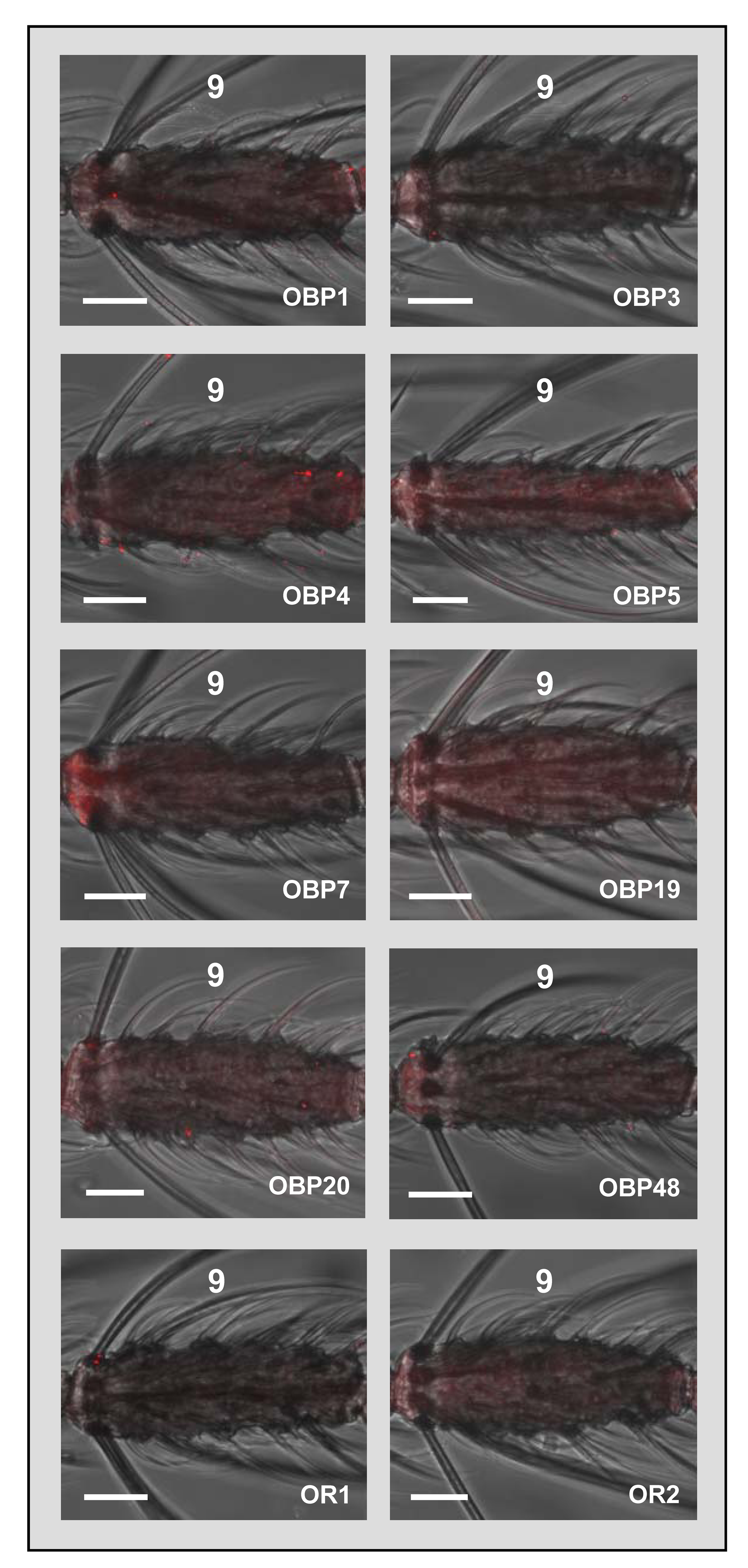

Supplement: Figure S1 — WM-FISH using AgOBP- or OR-specific DIG-labeled sense RNA probes and female A. gambiae antennae. No fluorescence labeling of cells (which would appear as red staining) was obtained with any of the sense probes tested. Only very weak background staining was obtained in some cases. The same (9th) flagellomere from different animals is shown. Pictures were taken using the same laser scanning microscope settings as the pictures shown in Figure1 for the OBP antisense RNA probes Scale bars: 20 µm. (TIF) [file pone.0069412.s001.tif]

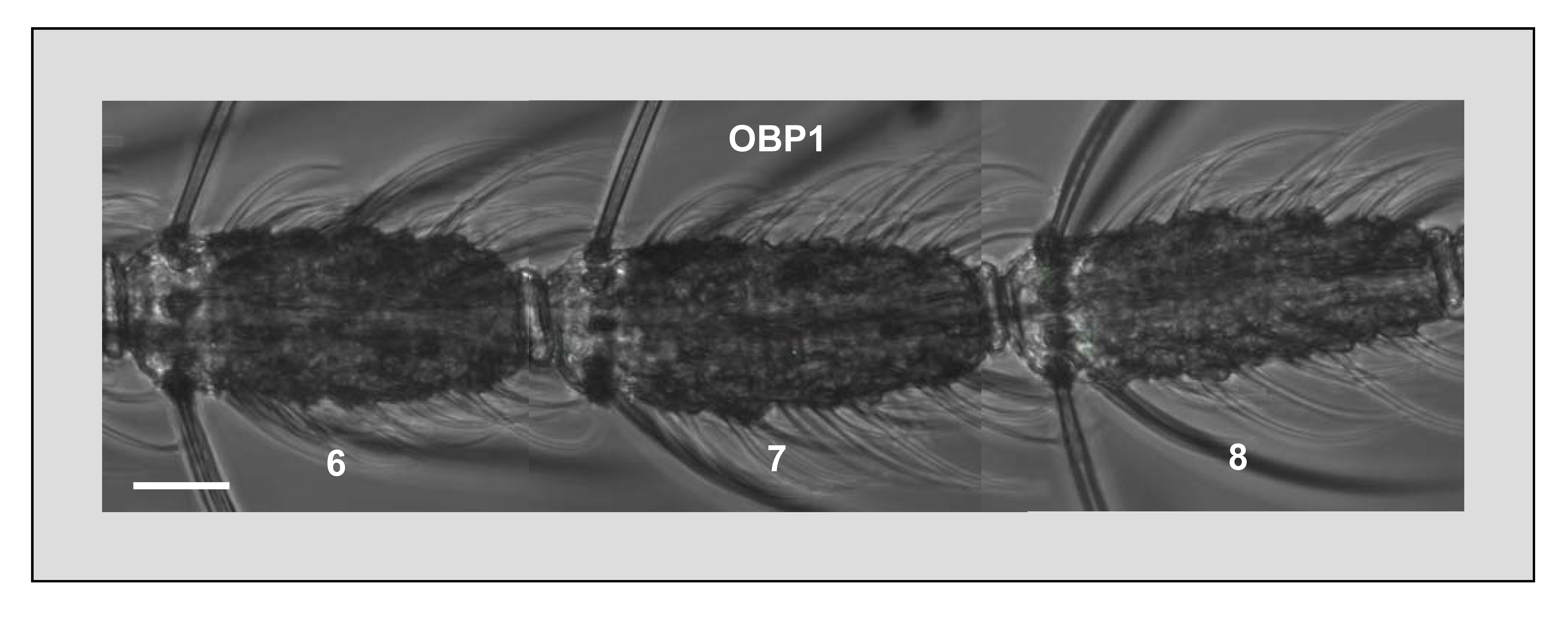

Supplement: Figure S2 — Whole mount preparations of female antennae were probed with pre-immune antiserum from animals, which were used to generate the OBP1-specific antiserum. No labeling of cells (which would be indicated by green color) was obtained with the pre-immune serum. The numbers of the flagellomere shown is indicated. Pictures were taken using the same laser scanning microscope settings as the pictures shown in Figure 6 for the OBP1 antiserum. Scale bars: 20 µm. (TIF) [file pone.0069412.s002.tif]
