# Supplementary material for: The Co-Expression Pattern of Odorant Binding Proteins and Olfactory Receptors Identify Distinct Trichoid Sensilla on the Antenna of the Malaria Mosquito Anopheles gambiae
Source: PLoS One. 2013 Jul 5;8(7):e69412. doi: 10.1371/journal.pone.0069412 (PMC3702612; doi:10.1371/journal.pone.0069412)
Supplement: Table S1 — AgOBPs and AgORs Gene Bank Accession numbers and nucleotide regions used as probes in whole mount fluorescence in situ hybridization experiments are indicated. (DOC) [file pone.0069412.s003.doc]

| **Gene** | **Database** | **Accession-Nr.** | **Nucleotide** |
| --- | --- | --- | --- |
|  |  |  |  |
| AgOBP1 | NCBI GeneBank | AF437884.1 | 89-523 |
| AgOBP3 | NCBI GeneBank | AF437886.1 | 124-585 |
| AgOBP4 | NCBI GeneBank | AF437887.1 | 75-524 |
| AgOBP5 | NCBI GeneBank | AF437888.1 | 89-550 |
| AgOBP7 | NCBI GeneBank | AF437890.1 | 203-664 |
| AgOBP19 | NCBI GeneBank | XM_313719.3 | 49-413 |
| AgOBP20 | NCBI GeneBank | XM_314108.4 | 321-683 |
| AgOBP48 | NCBI GeneBank | AY330175.1 | 1-123, 188-310, 384-740 |
|  |  |  |  |
| AgOR1 | NCBI GeneBank | XM_318674.1 | 1-1254 |
| AgOR2 | NCBI GeneBank | XM_310173.1 | 1-1137 |

**Table S1**. Sequences used for whole mount fluorescence *in situ* hybridization
